# Between Perfectly Critical and Fully Irregular: A Reverberating Model Captures and Predicts Cortical Spike Propagation

**DOI:** 10.1093/cercor/bhz049

**Published:** 2019-03-11

**Authors:** J Wilting, V Priesemann

**Affiliations:** 1 Max-Planck-Institute for Dynamics and Self-Organization, Am Faß berg 17, Göttingen, Germany; 2 Bernstein-Center for Computational Neuroscience, Göttingen, Germany

**Keywords:** balanced state, criticality, perturbations, timescales

## Abstract

Knowledge about the collective dynamics of cortical spiking is very informative about the underlying coding principles. However, even most basic properties are not known with certainty, because their assessment is hampered by spatial subsampling, i.e., the limitation that only a tiny fraction of all neurons can be recorded simultaneously with millisecond precision. Building on a novel, subsampling-invariant estimator, we fit and carefully validate a minimal model for cortical spike propagation. The model interpolates between two prominent states: asynchronous and critical. We find neither of them in cortical spike recordings across various species, but instead identify a narrow “reverberating” regime. This approach enables us to predict yet unknown properties from very short recordings and for every circuit individually, including responses to minimal perturbations, intrinsic network timescales, and the strength of external input compared to recurrent activation “thereby informing about the underlying coding principles for each circuit, area, state and task.

## Introduction

In order to understand how each cortical circuit or network processes its input, it would be desirable to first know its basic dynamical properties. For example, knowing which impact one additional spike has on the network (London et al. 2010) would give insight into the amplification of small stimuli (Douglas et al. 1995; Suarez et al. 1995; Miller 2016). Knowing how much of cortical activity can be attributed to external activation or internal activation (Reinhold et al. 2015) would allow to gauge how much of cortical activity is actually induced by stimuli, or rather internally generated, for example in the context of predictive coding (Rao and Ballard 1999; Clark 2013). Knowing the intrinsic network timescale (Murray et al. 2014) would inform how long stimuli are maintained in the activity and can be read out for short-term memory (Buonomano and Merzenich 1995; Wang 2002; Jaeger et al. 2007; Lim and Goldman 2013). However, not even these basic properties of cortical network dynamics are generally known with certainty.

In the past, insights about these network properties have been strongly hampered by the inevitable limitations of spatial subsampling, i.e., the fact that only a tiny fraction of all neurons can be recorded experimentally with millisecond precision. Such spatial subsampling fundamentally limits virtually any recording and hinders inferences about the collective response of cortical networks (Priesemann et al. 2009, 2014; Ribeiro et al. 2010, 2014; Levina and Priesemann 2017).

To describe network responses, two contradicting hypotheses have competed for more than a decade, and are the subjects of ongoing scientific debate: one hypothesis suggests that collective dynamics are “asynchronous-irregular” (AI) (Burns and Webb 1976; Softky and Koch 1993; Stein et al. 2005), i.e., neurons spike independently of each other and in a Poisson manner, which may reflect a balanced state (van Vreeswijk and Sompolinsky 1996; Brunel 2000). The other hypothesis proposes that neuronal networks operate at criticality (Beggs and Plenz 2003; Levina et al. 2007, 2009; Beggs and Timme 2012; Plenz and Niebur 2014; Tkačik et al. 2015; Humplik and Tkačik 2017; Muñoz 2018). Criticality is a particular state at a phase transition, characterized by high sensitivity and long-range correlations in space and time.

These hypotheses have distinct implications for the coding strategy of the brain. The typical balanced state minimizes redundancy (Atick 1992; Bell and Sejnowski 1997; van Hateren and van der Schaaf 1998; Hyvärinen and Oja 2000; Barlow 2012), supports fast network responses (van Vreeswijk and Sompolinsky 1996), and shows vanishing autocorrelation time or network timescale. In contrast, criticality in models optimizes performance in tasks that profit from extended reverberations of activity in the network (Bertschinger and Natschläger 2004; Haldeman and Beggs 2005; Kinouchi and Copelli 2006; Wang et al. 2011; Boedecker et al. 2012; Shew and Plenz 2013; Del Papa et al. 2017).

Surprisingly, there is experimental evidence for both AI and critical states in cortical networks, although both states are clearly distinct. Evidence for the AI state is based on characteristics of single-neuron spiking, resembling a Poisson process, i.e., exponential inter-spike interval (ISI) distributions and a Fano factor F close to unity (Burns and Webb 1976; Tolhurst et al. 1981; Vogels et al. 1989; Softky and Koch 1993; de Ruyter van Steveninck et al. 1997; Gur et al. 1997; Kara et al. 2000; Carandini 2004). Moreover, spike count cross-correlations (Ecker et al. 2010; Cohen and Kohn 2011) are small. In contrast, evidence for criticality was typically obtained from a population perspective instead, and assessed neuronal avalanches, i.e., spatio-temporal clusters of activity (Beggs and Plenz 2003; Pasquale et al. 2008; Priesemann et al. 2009; Friedman et al. 2012; Tagliazucchi et al. 2012; Shriki et al. 2013), whose sizes are expected to be power-law distributed if networks are critical (Bak et al. 1987). Deviations from power-laws, typically observed for spiking activity in awake animals (Bédard et al. 2006; Hahn et al. 2010; Ribeiro et al. 2010; Priesemann et al. 2014), were attributed to subsampling effects (Girardi-Schappo et al. 2013; Priesemann et al. 2009, 2013, 2014; Ribeiro et al. 2010, 2014; Levina and Priesemann 2017). Hence, different analysis approaches provided evidence for one or the other hypothesis about cortical dynamics.

**Figure 1.**
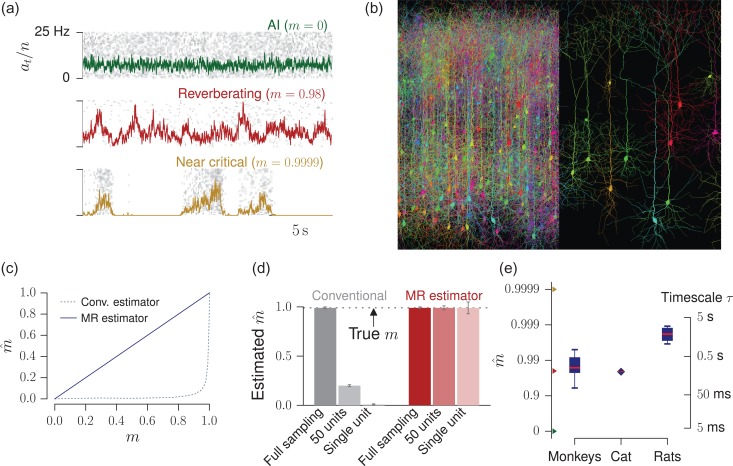
Reverberating versus critical and irregular dynamics under subsampling. (*a*) Raster plot and population rate $a_{t}$ for networks with different spike propagation parameters or neural efficacy m. They exhibit vastly different dynamics, which readily manifest in the population activity. (*b*) When recording spiking activity, only a small subset of all neurons can be sampled with millisecond precision. This spatial subsampling can hinder correct inference of collective properties of the whole network; figure created using TREES (Cuntz et al. 2010) and reproduced from Wilting and Priesemann (2018). (*c*) Estimated branching ratio $\hat{m}$ as a function of the simulated, true branching ratio m, inferred from subsampled activity (100 out of 10 000 neurons). While the conventional estimator misclassified m from this subsampled observation (gray, dotted line), the novel multistep regression (MR) estimator returned the correct values. (*d*) For a reverberating branching model with m= 0.98, the conventional estimator inferred $\hat{m}\;= 0.21$ or $\hat{m}\;= 0.002$ when sampling 50 or 1 units, respectively, in contrast to MR estimation, which returned the correct $\hat{m}$ even under strong subsampling. (*e*) Using the novel MR estimator, cortical network dynamics in monkey prefrontal cortex, cat visual cortex, and rat hippocampus consistently showed reverberating dynamics, with (median $\hat{m}\;= 0.98$ over all experimental sessions, boxplots indicate median/25–75%/0–100% over experimental sessions per species). These correspond to intrinsic network timescales between 80 ms and 2 s.

We here resolve the contradictory results about cortical dynamics, building on a subsampling-invariant approach presented in a companion study (Wilting and Priesemann 2018): (i) we establish an analytically tractable minimal model for *in vivo*-like activity, which can interpolate from AI to critical dynamics (Fig. 1*a*); (ii) we estimate the dynamical state of cortical activity based on a novel, subsampling-invariant estimator (Wilting and Priesemann 2018) (Figs. 1*b–d*); (iii) the model reproduces a number of dynamical properties of the network, which are experimentally accessible and enable us to validate our approach; (iv) we predict a number of yet unknown network properties, including the expected number of spikes triggered by one additional spike, the intrinsic network timescale, the distribution of the total number of spikes triggered by a single extra action potential, and the fraction of activation that can be attributed to afferent external input compared to recurrent activation in a cortical network.

## Material and Methods

We analyzed *in vivo* spiking activity from Macaque monkey prefrontal cortex during a short-term memory task (Pipa et al. 2009; Franke et al. 2010; Pröpper and Obermayer 2013), anesthetized cat visual cortex with no stimulus (Blanche and Swindale 2006; Blanche 2009), and rat hippocampus during a foraging task (Mizuseki et al. 2009a, 2009b) (Supp. 1). We compared the recordings of each experimental session to results of a minimal model of spike propagation, which is detailed in the following.

### Minimal Model of Spike Propagation

To gain an intuitive understanding of our mathematical approach, make a thought experiment in your favorite spiking network: apply one additional spike to an excitatory neuron, in analogy to the approach by (London et al. 2010). How does the network respond to that perturbation? As a first order approximation, one quantifies the number of spikes that are directly triggered *additionally* in all postsynaptic neurons. This number may vary from trial to trial, depending on the membrane potential of the postsynaptic neurons. However, what interests us most is m, the *mean number of spikes triggered by the one extra spike*. Any of these triggered spikes can in turn trigger spikes in their postsynaptic neurons in a similar manner, and thereby the perturbation may cascade through the system.

In the next step, assume that perturbations are started continuously at rate h, for example through afferent input from other brain areas or sensory modalities. Together, this leads to the mathematical framework of a branching model (Harris 1963; Heathcote 1965; Pakes 1971; Beggs and Plenz 2003; Haldeman and Beggs 2005; Ribeiro et al. 2010; Priesemann et al. 2013, 2014). This framework describes the number of active neurons $A_{t}$ in discrete time bins of length Δt. Here, Δt should reflect the propagation time of spikes between neurons. Formally, each spike i at the time bin t excites a random number $Y_{t,i}$ of postsynaptic spikes, on average $m=Y_{t,i}$. The activity $A_{t+1}$, i.e., the total number of spikes in the next time bin is then defined as the sum of the postsynaptic spikes of all current spikes $A_{t}$, as well as the input $h_{t}$:

(1) $$A_{t+1}=\overset{A_{t}}{\underset{i=1}{\sum }}Y_{t,i}+h_{t}.$$

This generic spiking model can generate dynamics spanning AI and critical states depending on the input (Zierenberg et al. 2018), and hence is well suited to probe network dynamics *in vivo* (see Supp. 3 for details). Most importantly, this framework enables us to infer m and other properties from the ongoing activity proper. Mathematical approaches to infer m are long known if the full network is sampled (Heyde and Seneta 1972; Wei 1991). Under subsampling, however, it is the novel estimator described in Wilting and Priesemann (2018) that for the first time allows an unbiased inference of m, even if only a tiny fraction of neurons is sampled.

A precise estimate of m is essential, because the dynamics of the model is mainly governed by m (Fig. 1*a*). Therefore, after inferring m, a number of quantities can be analytically derived, and others can be obtained by simulating a branching model, which is constrained by the experimentally measured m and the spike rate.

### Simulation

We simulated a branching model by mapping a branching process (Eq. (1) and Supp. 3) onto a random network of N= 10,000 neurons in the annealed disorder limit (Haldeman and Beggs 2005). An active neuron activated each of its κ= 4 postsynaptic neurons with probability p=m/κ. Here, the activated postsynaptic neurons were drawn randomly without replacement at each step, thereby avoiding that two different active neurons would both activate the same target neuron. The branching model is critical for m= 1 in the infinite-size limit, and subcritical (supercritical) for m< 1 (m> 1). We modeled input to the network at rate h by Poisson activation of each neuron at rate h/N. Subsampling (Priesemann et al. 2009) was applied to the model by sampling the activity of n neurons only, which were selected randomly before the simulation, and neglecting the activity of all other neurons. Thereby, instead of the full activity $A_{t}$, only the subsampled activity $a_{t}$ was considered for observation.

If not stated otherwise, simulations were run for $L{\;= 10}^{7}$ time steps (corresponding to ~11 h). Confidence intervals were estimated according to Wilting and Priesemann (2018) from B= 100 realizations of the model, both for simulation and experiments.

We compared the experimental recordings to three different models: AI, near-critical, and reverberating. All three models were set up to match the experiment in the number of sampled neurons n and firing rate $R=\langle a_{t}\rangle /(n\cdot \Delta t)$. The AI and near-critical models were set up with branching ratios of m= 0 or m= 0.9999, respectively. In addition, the reverberating model matched the recording in $m=\hat{m}$, where $\hat{m}$ was estimated from the recording using the novel subsampling-invariant estimator (see below). For all models, we chose a full network size of $N{\;= 10}^{4}$ and then determined the appropriate input h=RΔtN(1−m) in order to match the experimental firing rate. Exemplarily for the cat recording, which happened to represent the median $\hat{m}$, this yielded $\hat{m}\;= 0.98$, n= 50, and R= 7.25Hz. From these numbers, h= 290, h= 5.8 and h= 0.029 followed for the AI, reverberating, and near-critical models, respectively.

**Figure 2.**
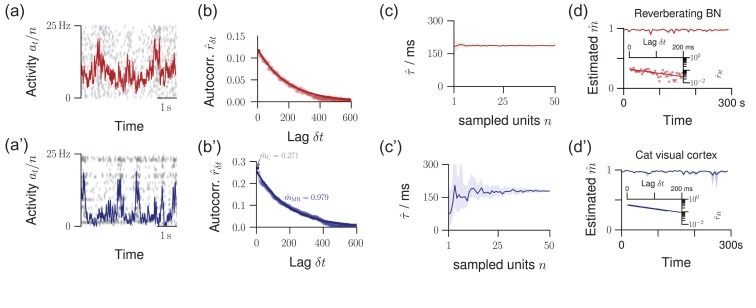
Validation of the model assumptions. The top row displays properties from a reverberating model, the bottom row spike recordings from cat visual cortex. (*a*/*a*’) Raster plot and population activity $a_{t}$ within bins of Δt= 4ms, sampled from n= 50 neurons. (*b*/*b*’) Multistep regression (MR) estimation from the subsampled activity (5 min recording). The predicted exponential relation $r_{\delta t}∼m^{\delta t/\Delta t}=\exp(-\delta t/\tau )$ provides a validation of the applicability of the model. The experimental data are fitted by this exponential with remarkable precision. (*c*/*c*’) When subsampling even further, MR estimation always returns the correct timescale $\hat{\tau }$ (or $\hat{m}$) in the model. In the experiment, this invariance to subsampling also holds, down to n≈10 neurons (shaded area: 16–84% confidence intervals estimated from 50 subsets of n neurons). (*d*/*d*’) The estimated branching parameter $\hat{m}$ for 59 windows of 5s length suggests stationarity of m over the entire recording (shaded area: 16–84% confidence intervals). The variability in $\hat{m}$ over consecutive windows was comparable for experimental recording and the matched model (p= 0.09, Levene test). Insets: exponential decay exemplified for one example window each.

In Fig. 2, the reverberating branching model was also matched to the length of the cat recording of 295 s. To test for stationarity, the cat recording and the reverberating branching model were split into 59 windows of 5 s each, before estimating m for each window. In Fig. 1*c*, subcritical and critical branching models with $N{\;= 10}^{4}$ and $A_{t}\;= 100$ were simulated, and n= 100 units sampled.

### **Subsampling-Invariant Estimation of** $\hat{m}$

Details on the analysis are found in Supp. 2. For each experimental recording, we collected the spike times of all recorded units (both single and multi-units) into one single train of population spike counts $a_{t}$, where $a_{t}$ denotes how many neurons spiked in the $t^{th}$ time bin Δt. If not indicated otherwise, we used Δt= 4ms, reflecting the propagation time of spikes from one neuron to the next.

From these experimental time series, we estimated $\hat{m}$ using the multistep regression (MR) estimator described in all detail in Wilting and Priesemann (2018). In brief, we calculated the linear regression slope $r_{k\Delta t}$, which describes the linear statistical dependence of $a_{t+k}$ upon $a_{t}$, for different time lags δt=kΔt with $k\;= 1,\ldots ,k_{\max}$. In our branching model, these slopes are expected to follow the relation $r_{\delta t}=b\cdot {\hat{m}}^{\delta t/\Delta t}(or\;r_{k\Delta t}=b\cdot {\hat{m}}^{k})$, where b is an unknown parameter that depends on the higher moments of the underlying process and the degree of subsampling. However, it can be partialled out, allowing for an estimation of m without further knowledge about b. Throughout this study, we chose $k_{\max}\;= 2500$ (corresponding to 10 s) for the rat recordings, $k_{\max}\;= 150$ (600 ms) for the cat recording, and *k*_max_ = 500 (2000 ms) for the monkey recordings, assuring that $k_{\max}\Delta t$ was always in the order of multiple intrinsic network timescales. In order to test for the applicability of a MR estimation, we used a set of conservative tests (Wilting and Priesemann 2018). The exponential relation can be rewritten as an exponential autocorrelation function $r_{\delta t}=bm^{\delta t/\Delta t}=\exp(\ln\;m\delta t/\Delta t)=\exp(-\delta t/\tau )$, where the intrinsic network timescale τ relates to m as m=exp(−Δt/τ). While the precise value of m depends on the choice of the bin size Δt and should only be interpreted together with the bin size (Δt= 4ms throughout this study), the intrinsic network timescale is independent of Δt. Therefore, we report both values in the following.

## Results

### Reverberating Spiking Activity *In Vivo*

We applied MR estimation to the binned population spike counts $a_{t}$ of the recorded neurons of each experimental session across three different species (see Methods). We identified a limited range of branching ratios *in vivo*: in the experiments $\hat{m}$ ranged from 0.963 to 0.998 (median = 0.98 , for a bin size of Δt=4ms), which is only a narrow window in the continuum from AI (m= 0) to critical (m= 1). For these values of found in cortical networks, the corresponding τ are between 100 ms and 2 s (median 247 ms, Figs 1*e* and S1). This clearly suggests that spiking activity *in vivo* is neither AI-like, nor consistent with a critical state. Instead, it is poised in a regime that, unlike critical or AI, does not maximize one particular property alone but may flexibly combine features of both (Wilting et al. 2018). Without a prominent characterizing feature, we name it the *reverberating regime*, stressing that activity reverberates (different from the AI state) at timescales of hundreds of milliseconds (different from a critical state, where they can persist infinitely).

### Validity of the Approach

There is a straight-forward verification of the validity of our phenomenological model: it predicts an exponential autocorrelation function $r_{\delta t}$ for the population activity $a_{t}$. We found that the activity in cat visual cortex (Fig. 2*a,a’*) is surprisingly well described by this exponential fit (Fig. 2*b,b’*). This validation holds to the majority of experiments investigated (14 out of 21, Fig. S1).

A second verification of our approach is based on its expected invariance under subsampling: We further subsampled the activity in cat visual cortex by only taking into account spikes recorded from a subset n′ out of all available n single units. As predicted (Fig. 2*c*), the estimates of $\hat{m}$, or equivalently of the intrinsic network timescale $\hat{\tau }$, coincided for any subset of single units if at least about five of the available 50 single units were evaluated (Fig. 2*c’*). Deviations when evaluating only a small subset of units most likely reflect the heterogeneity within cortical networks. Together, these results demonstrate that our approach returns consistent results when evaluating the activity of n≥5 neurons, which were available for all investigated experiments.

### Origin of the Activity Fluctuations

The fluctuations found in cortical spiking activity, instead of being intrinsically generated, could in principle arise from non-stationary input, which could in turn lead to misestimation of m (Priesemann and Shriki 2018). This is unlikely for three reasons: first, the majority of experiments passed a set of conservative tests that reject recordings that show any signature of common non-stationarities, as defined in (Wilting and Priesemann 2018). Second, recordings in cat visual cortex were acquired in absence of any stimulation, excluding stimulus-related non-stationarities. Third, when splitting the spike recording into short windows, the window-to-window variation of $\hat{m}$ in the recording did not differ from that of stationary *in vivo*-like reverberating models (p= 0.3, Fig. 2*d,d’*). For these reasons, the observed fluctuations in the estimates likely originate from the characteristic fluctuations of collective network dynamics within the reverberating regime.

### Timescales of the Network and Single Units

**Figure 3.**
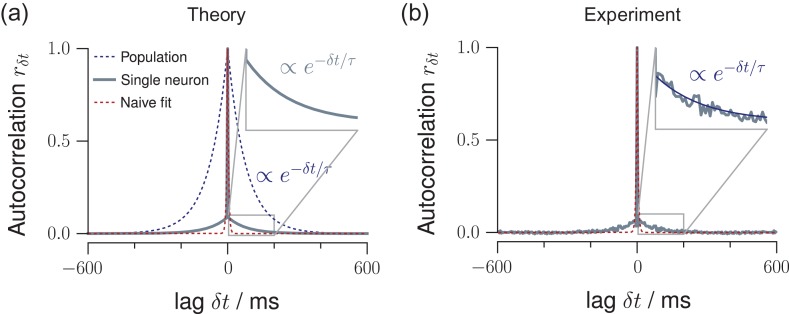
MR estimation and intrinsic network timescales. (*a*) In a branching model, the autocorrelation function of the population activity decays exponentially with an intrinsic network timescale τ (blue dotted line). In contrast, the autocorrelation function for single neurons shows a sharp drop from $r_{0}\;= 1$ at lag δt= 0 to the next lag $r_{\pm \Delta t}$ (gray solid line). We showed previously that this drop is a subsampling-induced bias. When ignoring the zero-lag value, the autocorrelation strength is decreased, but the exponential decay and even the value of the intrinsic network timescale τ of the network activity are preserved (inset). The red, dashed line shows a potential, naive exponential function, fitted to the single-neuron autocorrelation function (gray). This naive fit would return a much smaller τ. (*b*) The autocorrelation function of single-neuron activity recorded in cat visual cortex (gray) precisely resembles this theoretical prediction, namely a sharp drop and then an exponential decay (blue, inset), which persists over more than 100 ms. A naive exponential fit (red) to the single-neuron data would return an extremely short τ.

The dynamical state described by m directly relates to an exponential autocorrelation function with an intrinsic network timescale τ=−Δt/lnm. Exemplarily for the cat recording, m= 0.98 implies an intrinsic network timescale of τ= 188ms, with Δt= 4ms reflecting the spike propagation time from one neuron to the next. While the autocorrelation function of the full network activity is expected to show an exponential decay (Fig. 3*a*, blue), this is different for the autocorrelation of single neurons—the most extreme case of subsampling. We showed that subsampling can strongly decrease the absolute values of the autocorrelation function for any non-zero time lag (Fig. 3*a*, gray). This effect is typically interpreted as a lack of memory, because the autocorrelation of single neurons decays at the order of the bin size (Fig. 3*a*, red). However, ignoring the value at δt= 0, the floor of the autocorrelation function still unveils the exponential relation. Remarkably, the autocorrelation function of single units in cat visual cortex displayed precisely the shape predicted under subsampling (compare Fig. 3*a,b*).

### Established Methods are Biased to Identifying AI Dynamics

On the population level, networks with different m are clearly distinguishable (Fig. 1*a*). Surprisingly, single-neuron statistics, namely inter-spike interval (ISI) distributions, Fano factors, conventional estimation of m, and the autocorrelation strength $r_{\delta t}$, all returned signatures of AI activity regardless of the underlying network dynamics, and hence these single-neuron properties do not serve as a reliable indicator for the network’s dynamical state.

**Figure 4.**
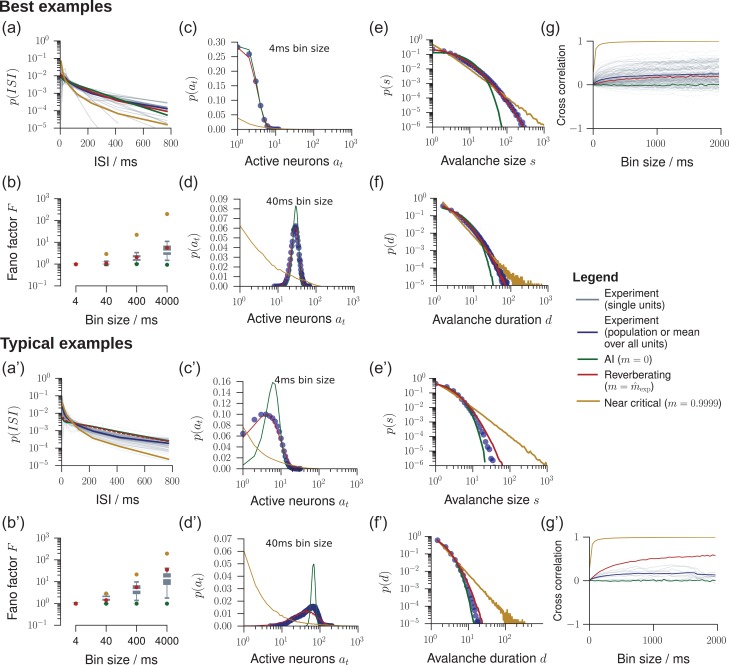
Model validation for *in vivo* spiking activity. We validated our model by comparing experimental results to predictions obtained from the *in vivo*-like, reverberating model, which was matched to the recording in the mean rate, inferred m, and number of recorded neurons. In general, the experimental results (gray or blue) were best matched by this reverberating model (red), compared to asynchronous-irregular (AI, green) and near-critical (yellow) models. From all experimental sessions, best examples (top) and typical examples (bottom) are displayed. For results from all experimental sessions see Figs S2–S8. (*a*/*a’*) Inter-spike-interval (ISI) distributions. (*b*/*b*’) Fano factors of single neurons for bin sizes between 4 ms and 4 s. (*c*/*c*’) Distribution of spikes per bin $p\left(a_{t}\right)$ at a bin size of 4 ms. (*d*/*d*’) Same as *c*/*c’* with a bin size of 40 ms. (*e*/*e*’) Avalanche size distributions p(s) for all sampled units. AI activity lacks large avalanches, near-critical activity produces power-law distributed avalanches, even under subsampling. (*f*/*f*’) Same as *e*/*e*’, but for the avalanche duration distributions p(d). (*g*/*g*’) Spike count cross-correlations ($r_{\mathrm{sc}}$) as a function of the bin size.

First, exponential ISI distributions are considered a strong indicator of Poisson-like firing. Surprisingly, the ISIs of single neurons in the *in vivo*-like branching model closely followed exponential distributions as well. The ISI distributions were almost indistinguishable for reverberating and AI models (Figs 4*a,a’* and S2). In fact, the ISI distributions are mainly determined by the mean firing rate. This result was further supported by coefficients of variation close to unity, as expected for exponential ISI distributions and Poisson firing (Fig. S2).

**Figure 5.**
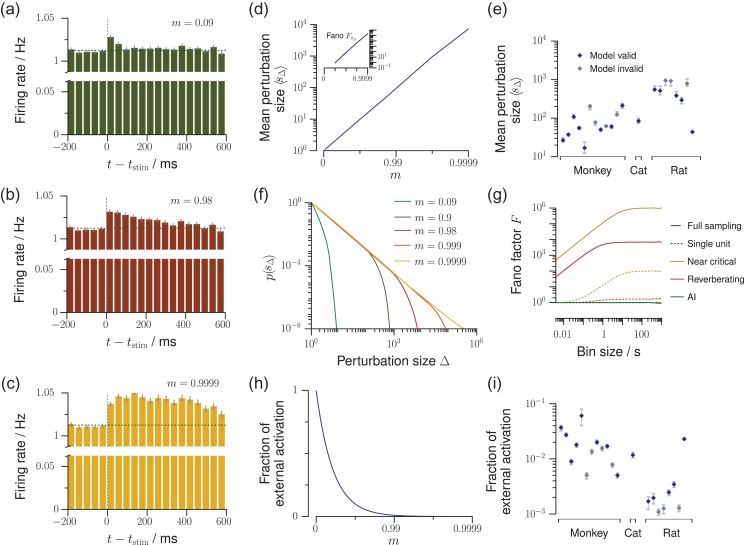
Predictions about network dynamics and propagation of perturbations. Using our *in vivo*-like, reverberating model, we can predict several network properties, which are yet very complicated or impossible to obtain experimentally. (*a***–***c*) In response to one single extra spike, a perturbation propagates in the network depending on the branching ratio m, and can be observed as a small increase of the average firing rate of the sampled neurons, here simulated for 500 trials (as in London et al. 2010). This increase of firing rate decays exponentially, with the decay time τ being determined by m. The perturbation *a* is rapidly quenched in the asynchronous-irregular state, *b* decays slowly over hundreds of milliseconds in the reverberating state, or *c* persists almost infinitely in the critical state. (*d*) The average perturbation size $s_{\Delta }$ and Fano factor $F_{s_{\Delta }}$ (inset) increase strongly with m. (*e*) Average total perturbation sizes predicted for each spike recording of mammalian cortex (errorbars: 5–95% confidence intervals). (*f*) Distribution $p\left(s_{\Delta }\right)$ of total perturbation sizes $s_{\Delta }$. The asynchronous-irregular models show approximately Poisson distributed, near-critical models power-law distributed perturbation sizes. (*g*) Bin size dependent Fano factors of the activity, here exemplarily shown for the asynchronous-irregular (m= 0, green), representative reverberating (m= 0.98, red), and near critical (m= 0.9999, yellow) models. While the directly measurable Fano factor of single neurons (dotted lines) underestimates the Fano factor of the whole network, the model allows to predict the Fano factor of the whole network (solid lines). (*h*) The fraction of the externally generated spikes compared to all spikes in the network strongly decreases with larger m. (*i*) Fraction of the externally generated spikes predicted for each spike recording of mammalian cortex (errorbars as in *e*).

Second, for both the AI and reverberating regime, the Fano factor F for single unit activity was close to unity, a hallmark feature of irregular spiking (Tolhurst et al. 1981; Vogels et al. 1989; Softky and Koch 1993; de Ruyter van Steveninck et al. 1997; Gur et al. 1997; Kara et al. 2000; Carandini 2004) (Fig. 5*g*, analytical result: Eq. (S9)). Hence it cannot serve to distinguish between these different dynamical states. When evaluating more units, or increasing the bin size to 4 s, the differences became more pronounced, but for experiments, the median Fano factor of single unit activity did not exceed F= 10 in any of the experiments, even in those with the longest reverberation (Figs 4*b*,*b’* and S3). In contrast, for the full network the Fano factor rose to $F\approx 10^{4}$ for the *in vivo*-like branching model and diverged when approaching criticality (Fig. 5*g*, analytical result: Eq. (S5)).

Third, conventional regression estimators (Heyde and Seneta 1972; Wei 1991) are biased towards inferring irregular activity, as shown before. Here, conventional estimation yielded a median of $\hat{m}\;= 0.057$ for single-neuron activity in cat visual cortex, in contrast to $\hat{m}\;= 0.954$ returned by MR estimation (Fig. S9).

Fourth, for the autocorrelation function of an experimental recording (Fig. 3*b*) the rapid decay of $r_{\delta t}$ prevails, and hence single-neuron activity appears uncorrelated in time.

### Cross-validation of Model Predictions

We compared the experimental results to an *in vivo*-like model, which was matched to each experiment only in the average firing rate, and in the inferred branching ratio $\hat{m}$. Remarkably, this *in vivo*-like branching model could predict statistical properties not only of single neurons (ISI and Fano factor, see above), but also pairwise and population properties, as detailed below. This prediction capability further underlines the usefulness of this simple model to approximate the default state of cortical dynamics.

First, the model predicted the activity distributions, $p\left(a_{t}\right)$, better than AI or critical models for the majority of experiments (15 out 21, Figs 4*c*,*d*,*c*’,*d*’, S5, and S6), both for the exemplary bin sizes of 4 ms and 40 ms. Hence, the branching models, which were only matched in their respective first moment of the activity distributions (through the rate) and first moment of the spreading behavior (through m), in fact approximated all higher moments of the activity distributions $p\left(a_{t}\right)$.

Likewise, the model predicted the distributions of neural avalanches, i.e., spatio-temporal clusters of activity (Figs 4*e*,*f*,*e*’,*f*’, S7, and S8). Characterizing these distributions is a classic approach to assess criticality in neuroscience (Beggs and Plenz 2003; Priesemann et al. 2014), because avalanche size and duration distributions, p(s) and p(d), respectively, follow power laws in critical systems. In contrast, for AI activity, they are approximately exponential (Priesemann and Shriki 2018). The matched branching models predicted neither exponential nor power law distributions for the avalanches, but very well matched the experimentally obtained distributions (compare red and blue in Figs 4*e*,*f*,*e*’,*f*’, S7, and S8). Indeed, model likelihood (Clauset et al. 2009) favored the *in vivo*-like branching model over Poisson and critical models for the majority experiments (18 out of 21, Fig. S7). Our results here are consistent with those of spiking activity in awake animals, which typically do not display power laws (Bédard et al. 2006; Ribeiro et al. 2010; Priesemann et al. 2014). In contrast, most evidence for criticality *in vivo*, in particular the characteristic power-law distributions, has been obtained from *coarse* measures of neural activity (LFP, EEG, BOLD; see Priesemann et al. 2014 and references therein).

Last, the model predicted the pairwise spike count cross-correlation $r_{\mathrm{sc}}$. In experiments, $r_{\mathrm{sc}}$ is typically between 0.01 and 0.25, depending on brain area, task, and most importantly, the analysis timescale (bin size) (Cohen and Kohn 2011). For the cat recording the model even correctly predicted the bin size dependence of $r_{\mathrm{sc}}$ from ${\bar{r}}_{\mathrm{sc}}\approx 0.004$ at a bin size of 4 ms (analytical result: Eq. (S12)) to ${\bar{r}}_{\mathrm{sc}}\approx 0.3$ at a bin size of 2 s (Fig. 4*g*). Comparable results were also obtained for some monkey experiments. In contrast, correlations in most monkey and rat recordings were smaller than predicted (Figs 4*g’* and S4). It is very surprising that the model correctly predicted the cross-correlation even in some experiments, as m was inferred only from the *temporal* structure of the spiking activity alone, whereas $r_{\mathrm{sc}}$ characterizes spatial dependencies.

Overall, by only estimating the effective synaptic strength m from the *in vivo* recordings, higher-order properties like avalanche size distributions, activity distributions and in some cases spike count cross-correlations could be closely matched using the generic branching model.

### The Dynamical State Determines Responses to Small Stimuli

After validating the model using a set of statistical properties that are well accessible experimentally, we now turn to making predictions about yet unknown properties, namely network responses to small stimuli. In the line of London et al. (2010), assume that on a background of spiking activity one single extra spike is triggered. This spike may in turn trigger new spikes, leading to a cascade of additional spikes $\Delta_{t}$ propagating through the network. A dynamical state with branching ratio m implies that *on average*, this perturbation decays with time constant τ=−Δt/logm. Similar to the approach in London et al. (2010), the evolution of the mean firing rate, averaged over a reasonable number of trials (here: 500) unveils the nature of the underlying spike propagation: depending on m, the rate excursions will last longer, the higher m (Figs 5*a*–c and S11). The perturbations are not deterministic, but show trial-to-trial variability which also increases with m (S11b).

Unless m> 1, the theory of branching models ensures that perturbations will die out eventually after a duration $d_{\Delta }$, having accumulated a total of $s_{\Delta }=\sum_{t\;= 1}^{d}\Delta_{t}$ extra spikes in total. This perturbation size $s_{\Delta }$ and duration $d_{\Delta }$ follow specific distributions (Harris 1963), which are determined by m: they are power law distributed in the critical state (m= 1), with a cutoff for any m< 1 (Figs 5*f* and S11*c*,*d*). These distributions imply a characteristic mean perturbation size $s_{\Delta }$ (Fig. 5*d*), which diverges at the critical point. The variability of the perturbation sizes is also determined by m and also diverges at the critical point (inset of Figs 5*d* and S11*e*).

Taken together, these results imply that the closer a neuronal network is to criticality, the more sensitive it is to external perturbations, and the better it can amplify small stimuli. At the same time, these networks also show larger trial-to-trial variability. For typical cortical networks, we found that the response to one single extra spike will on average comprise between 20 and 1000 additional spikes in total (Fig. 5*e*).

#### The Dynamical State Determines Network Susceptibility and Variability

Moving beyond single spike perturbations, our model gives precise predictions for the network response to continuous stimuli. If extra action potentials are triggered at rate h in the network, the network will again amplify these external activations, depending on m. Provided an appropriate stimulation protocol, this rate response could be measured and our prediction tested in experiments (Fig. S11*g*). The susceptibility ∂R/∂h diverges at the critical transition and is unique to a specific branching ratio m. We predict that typical cortical networks will amplify a small, but continuous input rate by about a factor fifty (Fig. S11*h*, red).

While the input and susceptibility determine the network’s mean activity, the network still shows strong rate fluctuations around this mean value. The magnitude of these fluctuations in relation to the mean can be quantified by the network Fano factor $F=\mathrm{Var}\left[A_{t}\right]/\langle A_{t}\rangle$ (Fig. 5*g*). This quantity cannot be directly inferred from experimental recordings, because the Fano factor of subsampled populations severely underestimates the network Fano factor, as shown before. We here used our *in vivo*-like model to obtain estimates of the network Fano factor: for a bin size of Δt= 4ms it is about F≈40 and rises to F≈4000 for bin sizes of several seconds, highlighting that network fluctuations probably are much stronger than one would naively assume from experimental, subsampled spiking activity.

#### Distinguishing Afferent and Recurrent Activation

Last, our model gives an easily accessible approach to solving the following question: given a spiking neuronal network, which fraction of the activity 〈A〉 is generated by recurrent activation from within the network, and which fraction can be attributed to external, afferent excitation *h*? The branching model readily provides an answer: the fraction of external activation is h/〈A〉= 1−m (Fig. 5*h*). In this framework, AI-like networks are completely driven by external input currents or noise, whereas reverberating networks generate a substantial fraction of their activity intrinsically. For the experiments investigated in this study, we inferred that between 0.1% and 7% of the activity are externally generated (median 2%, Fig. 5*i*).

While our model is quite simplistic given the complexity of neuronal network activity, keep in mind that “all models are wrong, but some are useful” (Box 1979). Here, the model has proven to provide a good first order approximation to a number of statistical properties of spiking activity and propagation in cortex. Hence, it promises insight into cortical function because (i) it relies on simply assessing spontaneous cortical activity, (ii) it does not require manipulation of cortex, (iii) it enables reasonable predictions about sensitivity, amplification, and internal and external activation, (iv) this analysis is possible in an area specific, task- and state-dependent manner as only short recordings are required for consistent results.

## Discussion

### Our Results Resolve Contradictions Between AI and Critical States

Our results for spiking activity *in vivo* suggest that network dynamics show AI-like statistics, because under subsampling the observed correlations are underestimated. In contrast, typical experiments that assessed criticality potentially overestimated correlations by sampling from overlapping populations (LFP, EEG) and thereby hampered a fine distinction between critical and subcritical states (Pinheiro Neto and Priesemann, in preparation). By employing for the first time a consistent, quantitative estimation, we provided evidence that *in vivo* spiking population dynamics reflects a reverberating regime, i.e., it operates in a narrow regime around m= 0.98. This result is supported by the findings by Dahmen et al. (2016): based on distributions of covariances, they inferred that cortical networks operate in a regime below criticality. Given the generality of our results across different species, brain areas, and cognitive states, our results suggest self-organization to this reverberating regime as a general organization principle for cortical network dynamics.

### The Reverberating Regime Combines Features of AI and Critical State

At first sight, $\hat{m}\;= 0.98$ of the reverberating regime may suggest that the collective spiking dynamics is very close to critical. Indeed, physiologically a Δm≈ 1.6% difference to criticality (m= 1) is small in terms of the effective synaptic strength. However, this apparently small difference in single unit properties has a large impact on the collective *dynamical* fingerprint and makes AI, reverberating, and critical states clearly distinct: for example, consider the sensitivity to a small input, i.e., the susceptibility $\chi =\partial R/\partial h=\frac{1}{1-m}$. The susceptibility diverges at criticality, making critical networks overly sensitive to input. In contrast, states with m≈0.98 assure sensitivity without instability. Because this has a strong impact on network dynamics and putative network function, finely distinguishing between dynamical states is both important and feasible even if the corresponding differences in effective synaptic strength (m) appear small.

We cannot ultimately rule out that cortical networks self-organize as close as possible towards criticality, the platonic ideal being impossible to achieve for example due to finite-size, external input, and refractory periods. Therefore, the reverberating regime might conform with quasi-criticality (Williams-García et al. 2014) or neutral theory (Martinello et al. 2017). However, we deem this unlikely for two reasons. First, in simulations of finite-size networks with external input, we could easily distinguish the reverberating regime from states with m= 0.9999 (Wilting and Priesemann 2018), which are more than one order of magnitude closer to criticality than any experiment we analyzed. Second, operating in a reverberating regime, which is between AI and critical, may combine the computational advantages of both states (Wilting et al. 2018): the reverberating regime enables rapid changes of computational properties by small parameter changes, keeps a sufficient safety-margin from instability to make seizures sufficiently unlikely (Priesemann et al. 2014), balances competing requirements (e.g., sensitivity and specificity (Gollo 2017)), and may carry short-term memory and allow to integrate information over limited, tunable timescales (Wang 2002; Boedecker et al. 2012). For these reasons, we consider the reverberating regime to be the explicit target state of self-organization. This is in contrast to the view of “as close to critical as possible,” which still holds criticality as the ideal target.

### More Complex Network Models

Cortical dynamics is clearly more complicated than a simple branching model. For example, heterogeneity of single-neuron morphology and dynamics, and non-trivial network topology likely impact population dynamics. However, we showed that *statistics* of cortical network activity are well approximated by a branching model. Therefore, we interpret branching models as a *statistical* approximation of spike propagation, which can capture a fair extent of the complexity of cortical dynamics. By using branching models, we draw on the powerful advantage of analytical tractability, which allowed for basic insight into dynamics and stability of cortical networks.

In contrast to the branching model, doubly stochastic processes (i.e., spikes drawn from an inhomogeneous Poisson distribution) failed to reproduce many statistical features (Fig. S10). We conjecture that the key difference is that doubly stochastic processes propagate the underlying firing rate instead of the actual spike count. Thus, propagation of the actual number of spikes (as e.g., in branching or Hawkes processes; Kossio et al. 2018), not some underlying firing rate, seems to be integral to capture the statistics of cortical spiking dynamics.

Our statistical model stands in contrast to *generative* models, which generate spiking dynamics by physiologically inspired mechanisms. One particularly prominent example are networks with balanced excitation and inhibition (van Vreeswijk and Sompolinsky 1996, 1997; Brunel 2000), which became a standard model of neuronal networks (Hansel and van Vreeswijk 2012). A balance of excitation and inhibition is supported by experimental evidence (Okun and Lampl 2008). Our statistical model reproduces statistical properties of such networks if one assumes that the excitatory and inhibitory contributions can be described by an effective excitation. In turn, the results obtained from the well-understood estimator can guide the refinement of generative models. For example, we suggest that network models need to be extended beyond the asynchronous-irregular state (Brunel 2000) to incorporate the network reverberations observed *in vivo*. Possible candidate mechanisms are increased coupling strength or inhomogeneous connectivity. Both have already been shown to induce rate fluctuations with timescales of several hundred milliseconds (Litwin-kumar and Doiron 2012; Ostojic 2014; Kadmon and Sompolinsky 2015).

Because of the assumption of uncorrelated, Poisson-like network firing, models that study single neurons typically assume that synaptic currents are normally distributed. Our results suggest that one should rather use input with reverberating properties with timescales of a few hundred milliseconds to reflect input from cortical neurons *in vivo*. This could potentially change our understanding of single-neuron dynamics, for example of their input-output properties.

### Deducing Network Properties from the Tractable Model

Using our analytically tractable model, we could predict and validate network properties, such as avalanche size and duration, ISI, or activity distributions. Given the experimental agreement with these predictions, we deduced further properties, which are impossible or difficult to assess experimentally and gave insight into more complex questions about network responses: How do perturbations propagate within the network, and How susceptible is the network to external stimulation?

One particular question we could address is the following: Which fraction of network activity is attributed to external or recurrent, internal activation? We inferred that about 98% of the activity is generated by recurrent excitation, and only about 2% originates from input or spontaneous threshold crossing. This result may depend systematically on the brain area and cognitive state investigated: For layer 4 of primary visual cortex in awake mice, Reinhold et al. (2015) concluded that the fraction of recurrent cortical excitation is about 72%, and cortical activity dies out with a timescale of about 12 ms after thalamic silencing. Their numbers agree perfectly well with our phenomenological model: a timescale of τ= 12ms implies that the fraction of recurrent cortical excitation is $m=e^{-\Delta t/\tau }\approx 72\%$, just as found experimentally. Under anesthesia, in contrast, they report timescales of several hundred milliseconds, in agreement with our results. These differences show that the fraction of external activation may strongly depend on cortical area, layer, and cognitive state. The novel estimator can in future contribute to a deeper insight into these differences, because it allows for a straight-forward assessment of afferent versus recurrent activation, simply from evaluating spontaneous spiking activity, without the requirement of thalamic or cortical silencing.

## Supplementary Material

supplementary Data
