# Supplementary material for: Between Perfectly Critical and Fully Irregular: A Reverberating Model Captures and Predicts Cortical Spike Propagation
Source: Cereb Cortex. 2019 Mar 11;29(6):2759–70. doi: 10.1093/cercor/bhz049 (PMC6519697; doi:10.1093/cercor/bhz049)
Supplement: supplementary Data [file bhz049_si.pdf]

Supplementary material for "Between perfectly critical and fully irregular: a reverberating model captures and predicts cortical spike propagation" by J. Wilting and V. Priesemann

Supp. 1 Experiments

We evaluated spike population dynamics from recordings in rats, cats and monkeys. The rat experimental protocols were approved by the Institutional Animal Care and Use Committee of Rutgers University (Mizuseki et al., 2009a,b). The cat experiments were performed in accordance with guidelines established by the Canadian Council for Animal Care (Blanche, 2009). The monkey experiments were performed according to the German Law for the Protection of Experimental Animals, and were approved by the Regierungspräsidium Darmstadt. The procedures also conformed to the regulations issued by the NIH and the Society for Neuroscience. The spike recordings from the rats and the cats were obtained from the NSF-founded CRCNS data sharing website (Blanche and Swindale, 2006, Blanche, 2009, Mizuseki et al., 2009a,b).

Rat experiments. In rats the spikes were recorded in CA1 of the right dorsal hippocampus during an open field task. We used the first two data sets of each recording group (ec013.527, ec013.528, ec014.277, ec014.333, ec015.041, ec015.047, ec016.397, ec016.430). The data-sets provided sorted spikes from 4 shanks (ec013) or 8 shanks (ec014, ec015, ec016), with 31 (ec013), 64 (ec014, ec015) or 55 (ec016) channels. We used both, spikes of single and multi units, because knowledge about the identity and the precise number of neurons is not required for the MR estimator. More details on the experimental procedure and the data-sets proper can be found in Mizuseki et al. (2009a,b).

Cat experiments. Spikes in cat visual cortex were recorded by Tim Blanche in the laboratory of Nicholas Swindale, University of British Columbia (Blanche, 2009). We used the data set pvc3, i.e. recordings of 50 sorted single units in area 18 (Blanche and Swindale, 2006). We used that part of the experiment in which no stimuli were presented, i.e., the spikes reflected spontaneous activity in the visual cortex of the anesthetized cat. Because of potential non-stationarities at the beginning and end of the recording, we omitted data before $25\,\mathrm{s}$ and after $320\,\mathrm{s}$ of recording. Details on the experimental procedures and the data proper can be found in Blanche (2009), Blanche and Swindale (2006).

Monkey experiments. The monkey data are the same as in Pipa et al. (2009), Priesemann et al. (2014). In these experiments, spikes were recorded simultaneously from up to 16 single-ended micro-electrodes ($\varnothing = 80\,\mu\mathrm{m}$) or tetrodes ($\varnothing = 96\,\mu\mathrm{m}$) in lateral prefrontal cortex of three trained macaque monkeys (M1: 6 kg ♀; M2: 12 kg ♂; M3: 8 kg ♀). The electrodes had impedances between 0.2 and $1.2\,\mathrm{M}\Omega$ at 1 kHz, and were arranged in a square grid with inter electrode distances of either 0.5 or 1.0 mm. The monkeys performed a visual short term memory task. The task and the experimental procedure is detailed in Pipa et al. (2009). We analyzed spike data from 12 experimental sessions comprising almost 12.000 trials (M1: 5 sessions; M2: 4 sessions; M3: 3 sessions). 6 out of 12 sessions were recorded with tetrodes. Spike sorting on the tetrode data was performed using a Bayesian optimal template matching approach as described in Franke et al. (2010) using the "Spyke Viewer" software (Pröpper and Obermayer, 2013). On the single electrode data, spikes were sorted with a multi-dimensional PCA method (Smart Spike Sorter by Nan-Hui Chen).

Supp. 2 Analysis

Temporal binning. For each recording, we collapsed the spike times of all recorded neurons into one single train of population spike counts a_t, where a_t denotes how many neurons spiked in the tth time bin Δt. If not indicated otherwise, we used $\Delta t = 4\,\mathrm{ms}$, reflecting the propagation time of spikes from one neuron to the next.

Multistep regression estimation of $\hat{m}$. From these time series, we estimated $\hat{m}$ using the MR estimator described in Wilting and Priesemann (2018). For $k = 1, \dots, k_{\mathrm{max}}$, we calculated the linear regression slope $r_{k\,\Delta t}$ for the linear statistical dependence of a_{t+k} upon a_t. From these slopes, we estimated $\hat{m}$ following the relation $r_{\delta t} = b \cdot \hat{m}^{\delta t/\Delta t}$, where b is an (unknown) parameter that depends on the higher moments of the underlying process and the degree of subsampling. However, for an estimation of m no further knowledge about b is required.

Throughout this study we chose $k_{\mathrm{max}} = 2500$ (corresponding to $10\,\mathrm{s}$) for the rat recordings, $k_{\mathrm{max}} = 150$ ($600\,\mathrm{ms}$) for the cat recording, and $k_{\mathrm{max}} = 500$ ($2000\,\mathrm{ms}$) for the monkey recordings, assuring that k_{max} was always in the order of multiple intrinsic network timescales (i.e., autocorrelation times).

In order to test for the applicability of a MR estimation, we used a set of conservative tests (Wilting and Priesemann, 2018), which found the expected exponential relation $r_{\delta t} = b\,m^{\delta t/\Delta t}$ in the majority of experimental recordings (14 out of 21, Fig. S1).

Avalanche size distributions. Avalanche sizes were determined similarly to the procedure described in Priesemann et al. (2009, 2014). Assuming that individual avalanches are separated in time, let $\{t_i\}$ indicate bins without activity, $a_{t_i} = 0$. The size s_i of one avalanche is defined by the integrated activity between two subsequent bins with zero activity:

$$s_i = \sum_{t=t_i}^{t_{i+1}} a_t. \tag{S1}$$

From the sample $\{s_i\}$ of avalanche sizes, avalanche size distributions $p(s)$ were determined using frequency counts. For illustration, we applied logarithmic binning, i.e. exponentially increasing bin widths for s.

For each experiments, these empirical avalanche size distributions were compared to avalanche size distributions obtained in a similar fashion from three different matched models (see below for details). Model likelihoods $l(\{s_i\}) \mid m)$ for all three models were calculated following Clauset et al. (2009), and we considered the likelihood ratio to determine the most likely model based on the observed data.

ISI distributions, Fano factors and spike count cross-correlations. For each experiment and corresponding reverberating branching model (subsampled to a single unit), ISI distributions were estimated by frequency counts of the differences between subsequent spike times for each channel.

We calculated the single unit Fano factor $F = \mathrm{Var}[a_t]/\langle a_t \rangle$ for the binned activity a_t of each single unit, with the bin sizes indicated in the respective figures. Likewise, single unit Fano factors for the reverberating branching models were calculated from the subsampled and binned time series.

From the binned single unit activities a_t^1 and a_t^2 of two units, we estimated the spike count cross correlation $r_{\mathrm{sc}} = \mathrm{Cov}(a_t^1, a_t^2)/\sigma_{a_t^1}\sigma_{a_t^2}$. The two samples a_t^1 and a_t^2 for the reverberating branching models were obtained by sampling two randomly chosen neurons.

Supp. 3 Branching processes

In a branching process (BP) with immigration (Harris, 1963, Heathcote, 1965, Pakes, 1971) each unit i produces a random number $y_{t,i}$ of units in the subsequent time step. Additionally, in each time step a random number h_t of units immigrates into the system (drive). Mathematically, BPs are defined as follows (Harris, 1963, Heathcote, 1965): Let $y_{t,i}$ be independently and identically

distributed non-negative integer-valued random variables following a law Y with mean $m = \langle Y \rangle$ and variance $\sigma^2 = \mathrm{Var}[Y]$. Further, Y shall be non-trivial, meaning it satisfies $\mathrm{P}[Y=0] > 0$ and $\mathrm{P}[Y=0] + \mathrm{P}[Y=1] < 1$. Likewise, let h_t be independently and identically distributed non-negative integer-valued random variables following a law H with mean rate $h = \langle H \rangle$ and variance $\xi^2 = \mathrm{Var}[H]$. Then the evolution of the BP A_t is given recursively by

$$A_{t+1} = \sum_{i=1}^{A_t} y_{t,i} + h_t, \tag{S2}$$

i.e. the number of units in the next generation is given by the offspring of all present units and those that were introduced to the system from outside.

The stability of BPs is solely governed by the mean offspring m. In the subcritical state, $m < 1$, the population converges to a stationary distribution A_∞ with mean $\langle A_\infty \rangle = h/(1-m)$. At criticality ($m = 1$), A_t asymptotically exhibits linear growth, while in the supercritical state ($m > 1$) it grows exponentially.

We will now derive results for the mean, variance, and Fano factor of subcritical branching processes. Following previous results, taking expectation values of both sides of Eq. (S2) yields $\langle A_{t+1} \rangle = m\langle A_t \rangle + h$. Because of stationarity $\langle A_{t+1} \rangle = \langle A_t \rangle = \langle A_\infty \rangle$ and the mean activity is given by

$$\langle A_\infty \rangle = \frac{h}{1-m}. \tag{S3}$$

In order to derive an expression for the variance of the stationary distribution, observe that by the theorem of total variance, $\mathrm{Var}[A_{t+1}] = \langle \mathrm{Var}[A_{t+1} \mid A_t] \rangle + \mathrm{Var}[\langle A_{t+1} \mid A_t \rangle]$, where $\langle \cdot \rangle$ denotes the expected value, and $A_{t+1} \mid A_t$ conditioning the random variable A_{t+1} on A_t. Because A_{t+1} is the sum of independent random variables, the variances also sum: $\mathrm{Var}[A_{t+1} \mid A_t] = \sigma^2 A_t + \xi^2$. Using the previous result for $\langle A_\infty \rangle$ one then obtains

$$\mathrm{Var}[A_{t+1}] = \xi^2 + \sigma^2 \frac{h}{1-m} + \mathrm{Var}[mA_t + h] = \xi^2 + \sigma^2 \frac{h}{1-m} + m^2 \mathrm{Var}[A_t].$$

Again, in the stationary distribution $\mathrm{Var}[A_{t+1}] = \mathrm{Var}[A_t] = \mathrm{Var}[A_\infty]$ which yields

$$\mathrm{Var}[A_\infty] = \frac{1}{1-m^2}\left(\xi^2 + \sigma^2 \frac{h}{1-m}\right), \tag{S4}$$

The Fano factor $F_{A_t} = \mathrm{Var}[A_t] \,/\, \langle A_t \rangle$ is easily computed from (S3) and (S4):

$$F_{A_t} = \frac{\xi^2}{h(1+m)} + \frac{\sigma^2}{1-m^2}. \tag{S5}$$

Interestingly, the mean rate, variance, and Fano factor all diverge when approaching criticality (given a constant input rate h): $\langle A_\infty \rangle \to \infty$, $\quad \mathrm{Var}[A_\infty] \to \infty$, and $F_{A_t} \to \infty$ as $m \to 1$.

These results were derived without assuming any particular law for Y or H. Although the limiting behavior of BPs does not depend on it (Harris, 1963, Heathcote, 1965, Pakes, 1971), fixing particular laws allows to simplify these expressions further.

We here chose Poisson distributions with means m and h for Y and H respectively: $y_{t,i} \sim \mathrm{Poi}(m)$ and $h_t \sim \mathrm{Poi}(h)$. We chose these laws for two reasons: (1) Poisson distributions allow for non-trivial offspring distributions with easy control of the branching ratio m by only one parameter. (2) For the brain, one might assume that each neuron is connected to k postsynaptic neurons, each of which is excited with probability p, motivating a binomial offspring distribution with mean $m = k\,p$. As in cortex k is typically large and p is typically small, the Poisson limit is a reasonable approximation. Choosing these distributions, the variance and Fano factor become

$$\begin{aligned} \mathrm{Var}[A_t] &= h \,/\, ((1-m)^2(1+m)), \\ F_{A_t} &= 1 \,/\, (1-m^2). \end{aligned} \tag{S6}$$

Both diverge when approaching criticality ($m = 1$).

Supp. 4 Subsampling

A general notion of subsampling was introduced in Wilting and Priesemann (2018). The subsampled time series a_t is constructed from the full process A_t based on the three assumptions: (i) The sampling process does not interfere with itself, and does not change over time. Hence the realization of a subsample at one time does not influence the realization of a subsample at another time, and the conditional *distribution* of $(a_t|A_t)$ is the same as $(a_{t'}|A_{t'})$ if $A_t = A_{t'}$.

However, even if $A_t = A_{t'}$, the subsampled a_t and $a_{t'}$ do not necessarily take the same value. (ii) The subsampling does not interfere with the evolution of A_t, i.e. the process evolves independent of the sampling. (iii) *On average* a_t is proportional to A_t up to a constant term, $\langle a_t \mid A_t \rangle = \alpha A_t + \beta$.

In the spike recordings analyzed in this study, the states of a subset of neurons are observed by placing electrodes that record the activity of the same set of neurons over the entire recording. This implementation of subsampling translates to the general definition in the following manner: If n out of all N neurons are sampled, the probability to sample a_t active neurons out of the actual A_t active neurons follows a hypergeometric distribution, $a_t \sim \mathrm{Hyp}(N, n, A_t)$. As $\langle a_t \mid A_t = j \rangle = j n / N$, this representation satisfies the mathematical definition of subsampling with $\alpha = n/N$. Choosing this special implementation of subsampling allows to derive predictions for the Fano factor under subsampling and the spike count cross correlation. First, evaluate $\mathrm{Var}[a_t]$ further in terms of A_t:

$$
\begin{aligned}
\mathrm{Var}[a_t] &= \langle \mathrm{Var}[a_t \mid A_t] \rangle + \mathrm{Var}[\langle a_t \mid A_t \rangle] \\
&= n \langle \frac{A_t}{N} \frac{N - A_t}{N} \frac{N-n}{N-1} \rangle + \mathrm{Var}[\frac{n}{N} A_t] \\
&= \frac{1}{N} \frac{n}{N} \frac{N-n}{N-1} \left(N \langle A_t \rangle - \langle A_t^2 \rangle \right) + \frac{n^2}{N^2} \mathrm{Var}[A_t] \\
&= \frac{n}{N^2} \frac{N-n}{N-1} \left(N \langle A_t \rangle - \langle A_t \rangle^2 \right) + \left(\frac{n^2}{N^2} - \frac{n}{N^2} \frac{N-n}{N-1} \right) \mathrm{Var}[A_t]. \qquad \text{(S7)}
\end{aligned}
$$

This expression precisely determines the variance $\mathrm{Var}[a_t]$ under subsampling from the properties $\langle A_t \rangle$ and $\mathrm{Var}[A_t]$ of the full process, and from the parameters of subsampling n and N. We now show that the Fano factor approaches and even falls below unity under strong subsampling, regardless of the underlying dynamical state m. In the limit of strong subsampling ($n \ll N$) Eq. (S7) yields:

$$
\mathrm{Var}[a_t] \approx \frac{n}{N^2} \left(N \langle A_t \rangle - \langle A_t \rangle^2 \right) + \frac{n^2 - n}{N^2} \mathrm{Var}[A_t]. \qquad \text{(S8)}
$$

Hence the subsampled Fano factor is given by

$$F_{a_t} = \frac{\mathrm{Var}[a_t]}{\langle a_t \rangle} \approx 1 - \frac{\langle A_t \rangle}{N} + \frac{n-1}{N}\frac{\mathrm{Var}[A_t]}{\langle A_t \rangle} = 1 - \frac{\langle A_t \rangle - (n-1)F_{A_t}}{N}. \tag{S9}$$

Interestingly, when sampling a single unit ($n = 1$) the Fano factor of that unit becomes completely independent of the Fano factor of the full process:

$$F_{a_t} = 1 - \langle A_t \rangle / N = 1 - \langle a_t \rangle / n = 1 - R, \tag{S10}$$

where $R = \langle a_t \rangle / n$ is the mean rate of a single unit.

Based on this implementation of subsampling, we derived analytical results for the cross-correlation between the activity of two units on the time scale of one time step. The pair of units is here represented by two independent samplings a_t and $\tilde{a}(t)$ of a BP A_t with $n = 1$, i.e. each represents one single unit. Because both samplings are drawn from identical distributions, their variances are identical and hence the correlation coefficient is given by $r_{\mathrm{sc}} = \mathrm{Cov}(a_t, \tilde{a}(t))\,/\mathrm{Var}[a_t]$. Employing again the law of total expectation and using the independence of the two samplings, this can be evaluated:

$$\mathrm{Cov}(a_t, \tilde{a}(t)) = \langle \langle a_t\, \tilde{a}(t) \,|\, A_t \rangle \rangle_{A_t} - \langle \langle a_t \,|\, A_t \rangle \rangle^2_{A_t} = \frac{1}{N^2}\mathrm{Var}[A_t], \tag{S11}$$

with the first inner expectation being taken over the joint distribution of a_t and $\tilde{a}(t)$. Using Eq. (S8), one easily obtains

$$r_{\mathrm{sc}} = \frac{\mathrm{Var}[A_t]}{N\langle A_t \rangle - \langle A_t \rangle^2} = \frac{F_{A_t}}{N - \langle A_t \rangle} = \frac{F_{A_t}}{N\,(1-R)} \tag{S12}$$

with the mean single unit rate $R = \langle A_t \rangle / N$. For subcritical systems, the Fano factor F_{A_t} is much smaller than N, and the rate is typically much smaller than 1. Therefore, the cross-correlation between single units is typically very small.

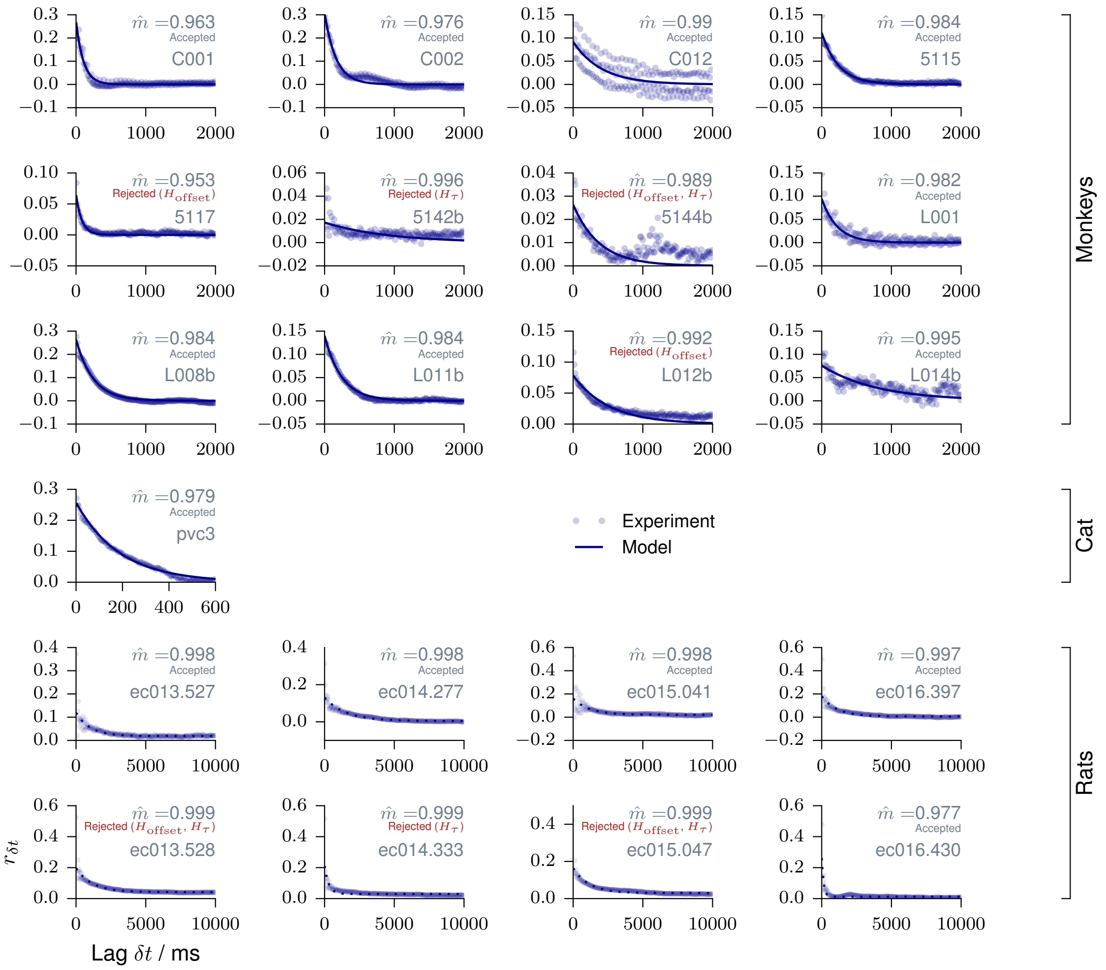

Figure S1: MR estimation for individual recording sessions. Reproduced from Wilting and Priesemann (2018). MR estimation is shown for every individual animal. The consistency checks are detailed in Wilting and Priesemann (2018). Data from monkey were recorded in prefrontal cortex during an working memory task. The third panel shows a oscillation of r_k with a frequency of 50 Hz, corresponding to measurement corruption due to power supply frequency. Data from anesthetized cat were recorded in primary visual cortex. Data from rat were recorded in hippocampus during a foraging task. In addition to a slow exponential decay, the slopes r_k show the ϑ-oscillations of 6 – 10 Hz present in hippocampus.

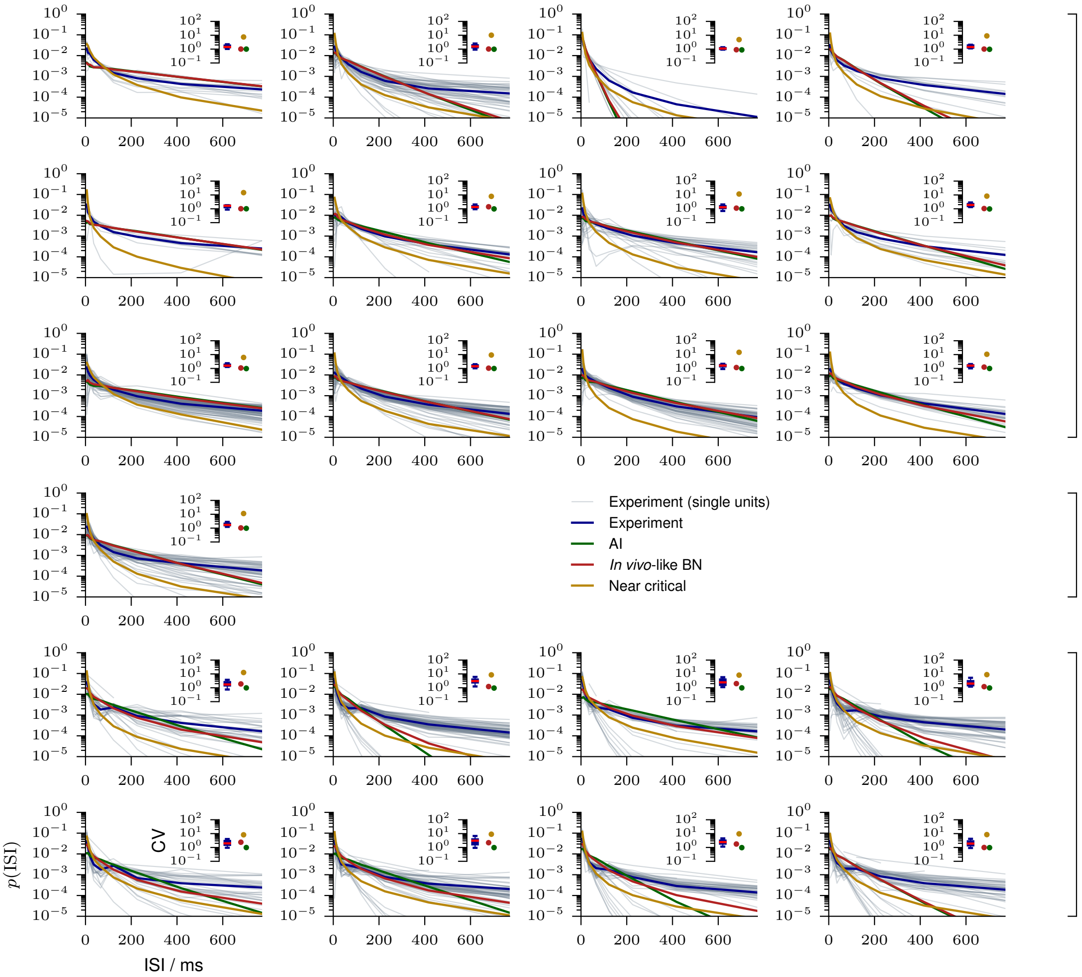

Figure S2: Interspike interval distribution for individual recording sessions. Interspike interval (ISI) distributions are shown for individual units of each recording (gray), for the average over units of each recording (blue), as well as for the matched models, either AI (green), *in vivo*-like (red), or near critical (yellow). The insets show the corresponding coefficients of variation (CV). For every experiment AI and *in vivo*-like models are virtually indistinguishable by the ISI distributions.

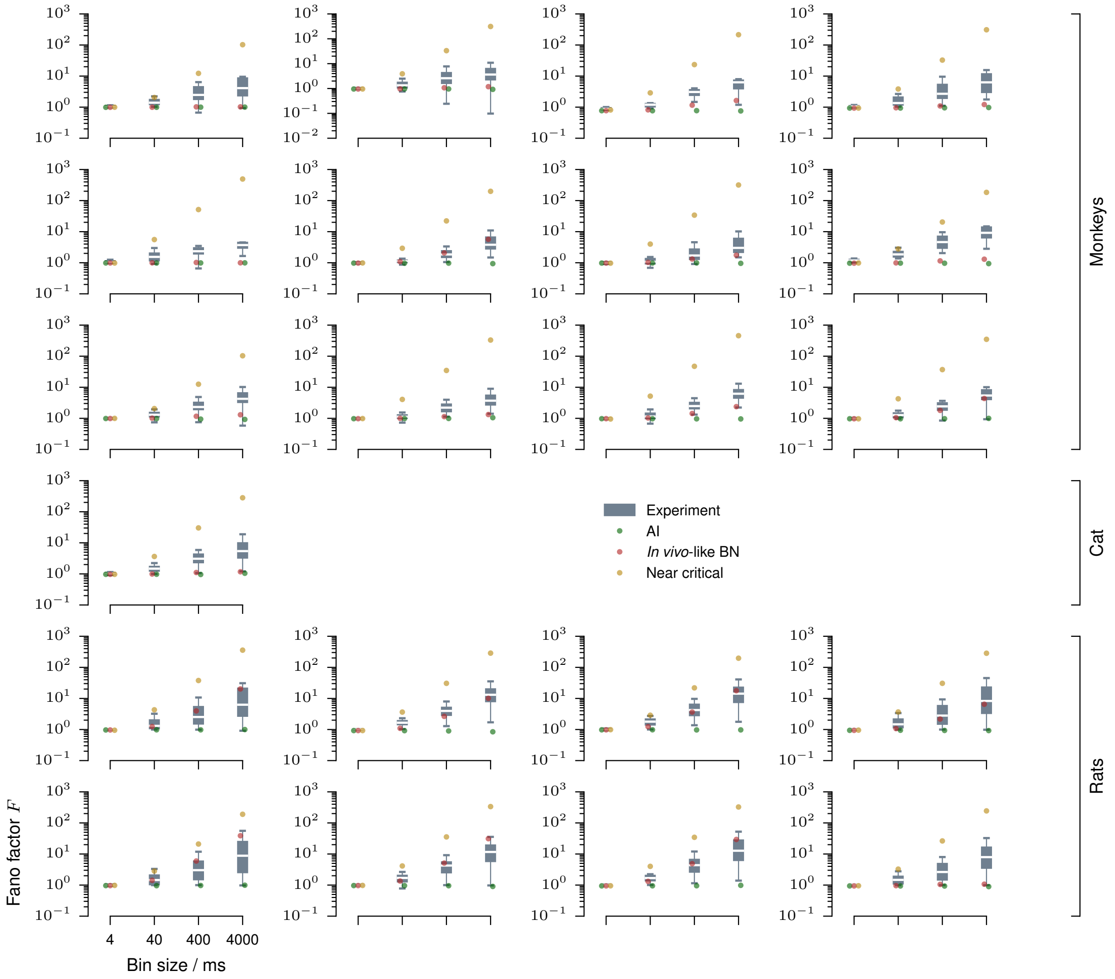

FIGURE S3: Fano factors for individual recording sessions. Fano factors are shown for individual single or multi units of every recording (gray boxplots, median / 25% – 75%, 2.5% – 97.5%), as well as for the matched models, either AI (green), *in vivo*-like (red), or near critical (yellow).

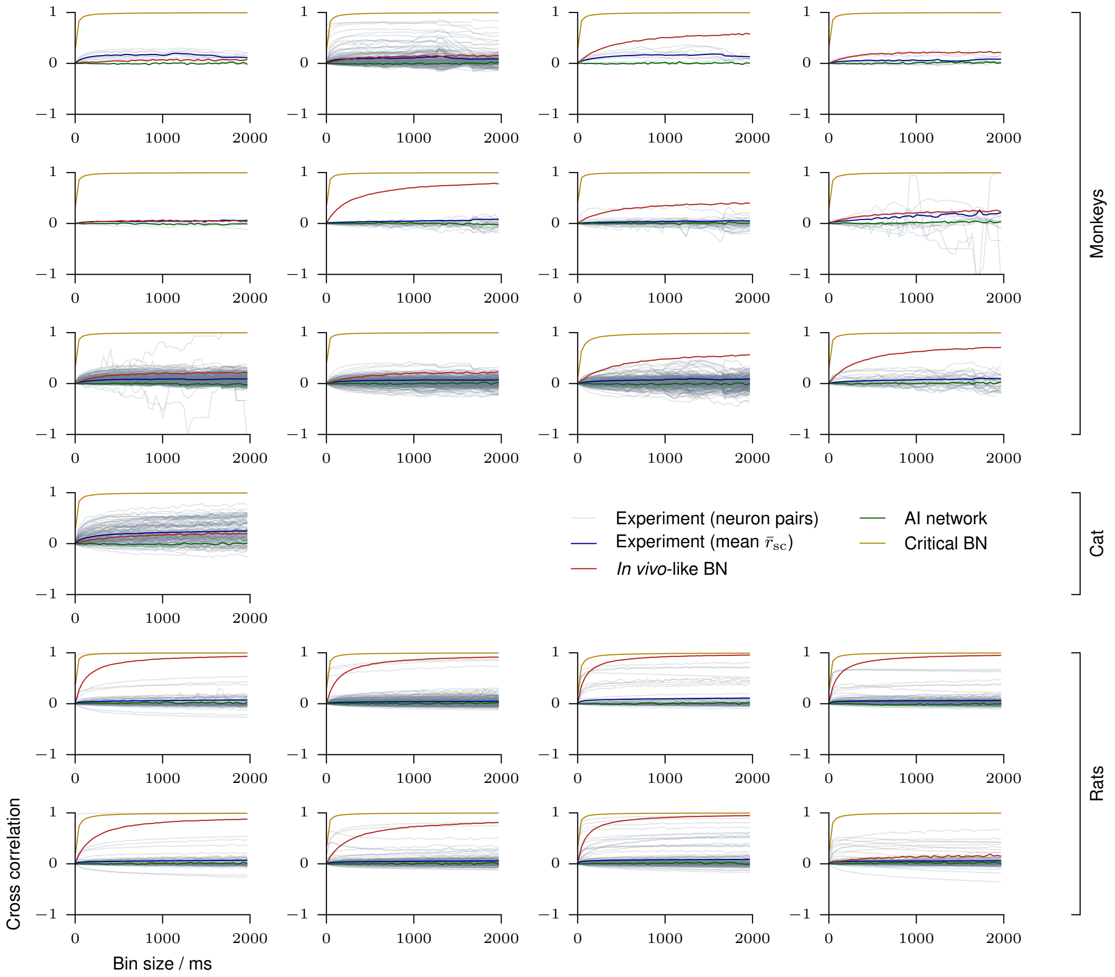

Figure S4: Cross correlations for individual recording sessions. Spike count cross correlations (r_{sc}) are shown for every neuron pair (gray) and the ensemble average (blue) of each recording, for bin sizes from 1 ms to 2s. Cross correlations are also shown for the matched models, either AI (green), *in vivo*-like (red), or near critical (yellow).

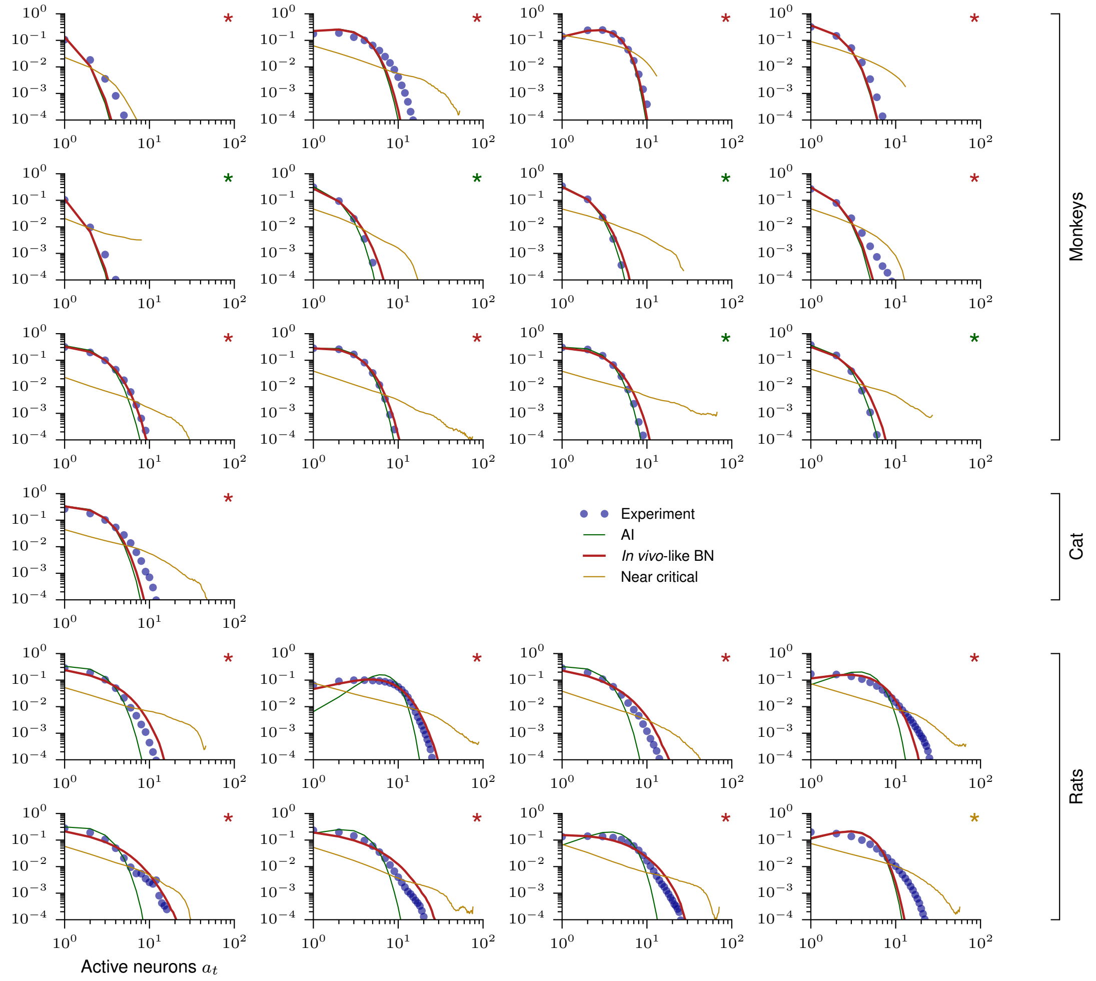

FIGURE S5: Activity distributions (4 ms bin size). Activity distributions are shown for every recording for a bin size of 4 ms (blue). Activity distributions for the matched models, either AI (green), *in vivo*-like (red), or near critical (yellow) are also shown. The color of the asterisk indicates which of the three models yielded the highest likelihood for the data following Clauset et al. (2009).

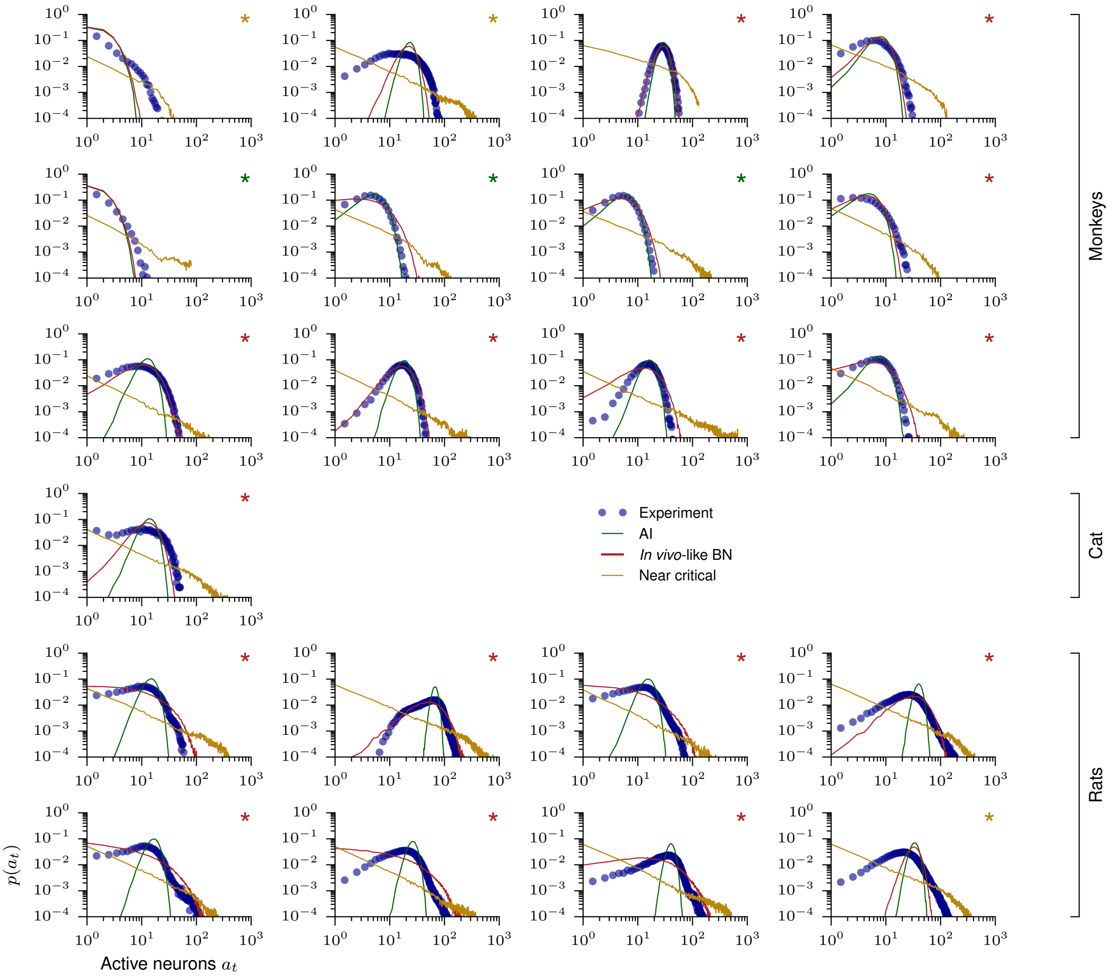

Figure S6: Activity distributions (40 ms bin size). Activity distributions are shown for every recording, for a bin size of 40 ms (blue). Activity distributions for the matched models, either AI (green), *in vivo*-like (red), or near critical (yellow) are also shown. The color of the asterisk indicates which of the three models yielded the highest likelihood for the data following Clauset et al. (2009).

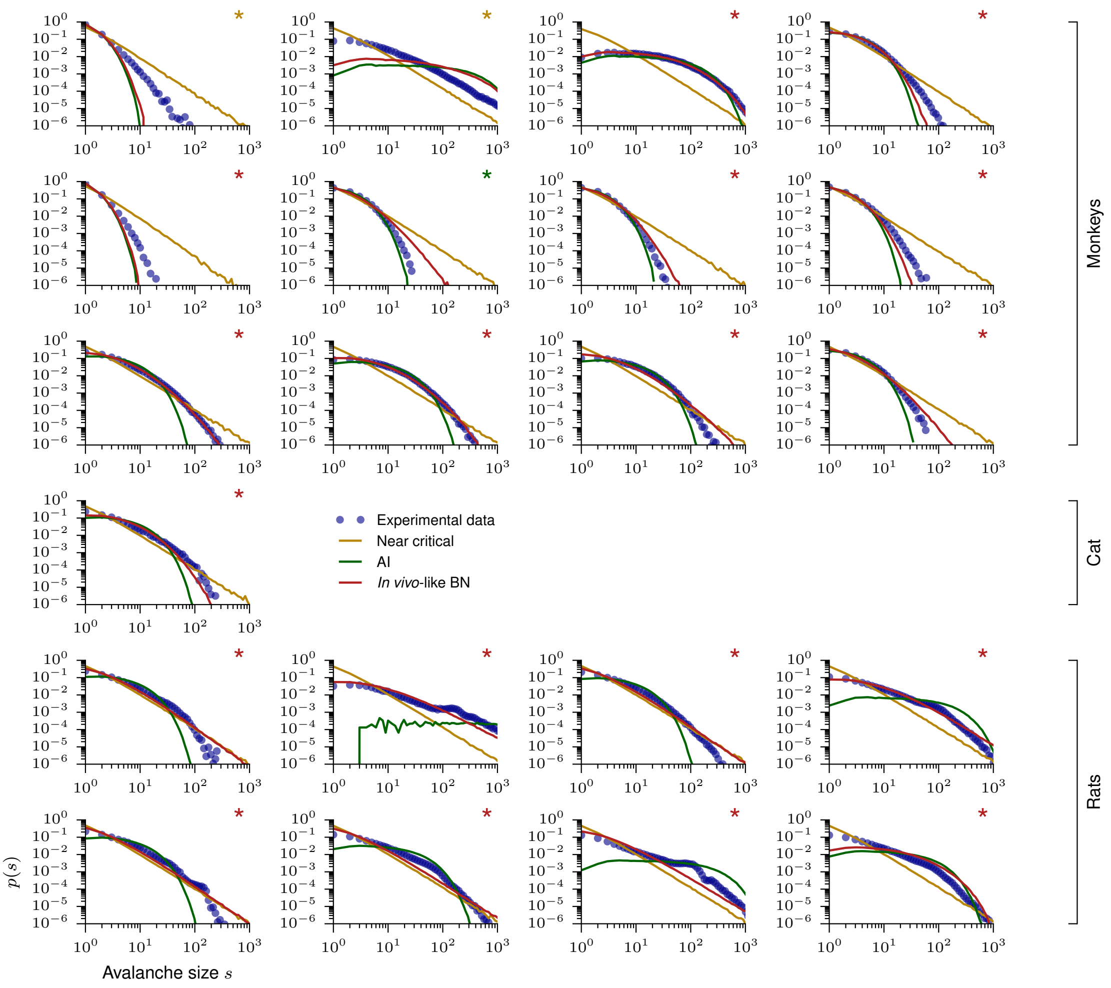

Figure S7: Avalanche size distribution for individual recording sessions. Avalanche size distributions are shown for every recording (blue) and for matched models, either AI (green), *in vivo*-like (red), or near critical (yellow). The color of the asterisk indicates which of the three models yielded the highest likelihood for the data following Clauset et al. (2009).

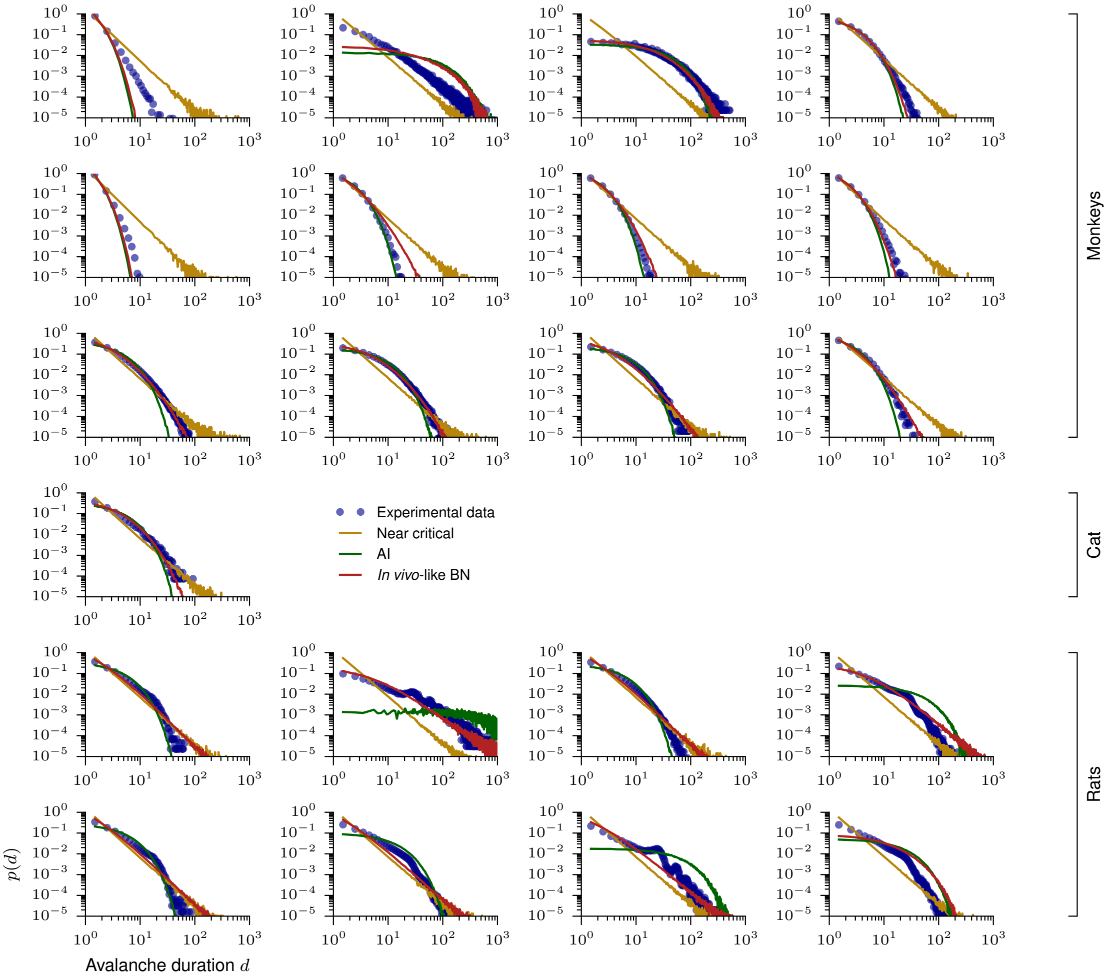

Figure S8: Avalanche duration distribution for individual recording sessions. Avalanche duration distributions are shown for every recording (blue) and for matched models, either AI (green), *in vivo*-like (red), or near critical (yellow).

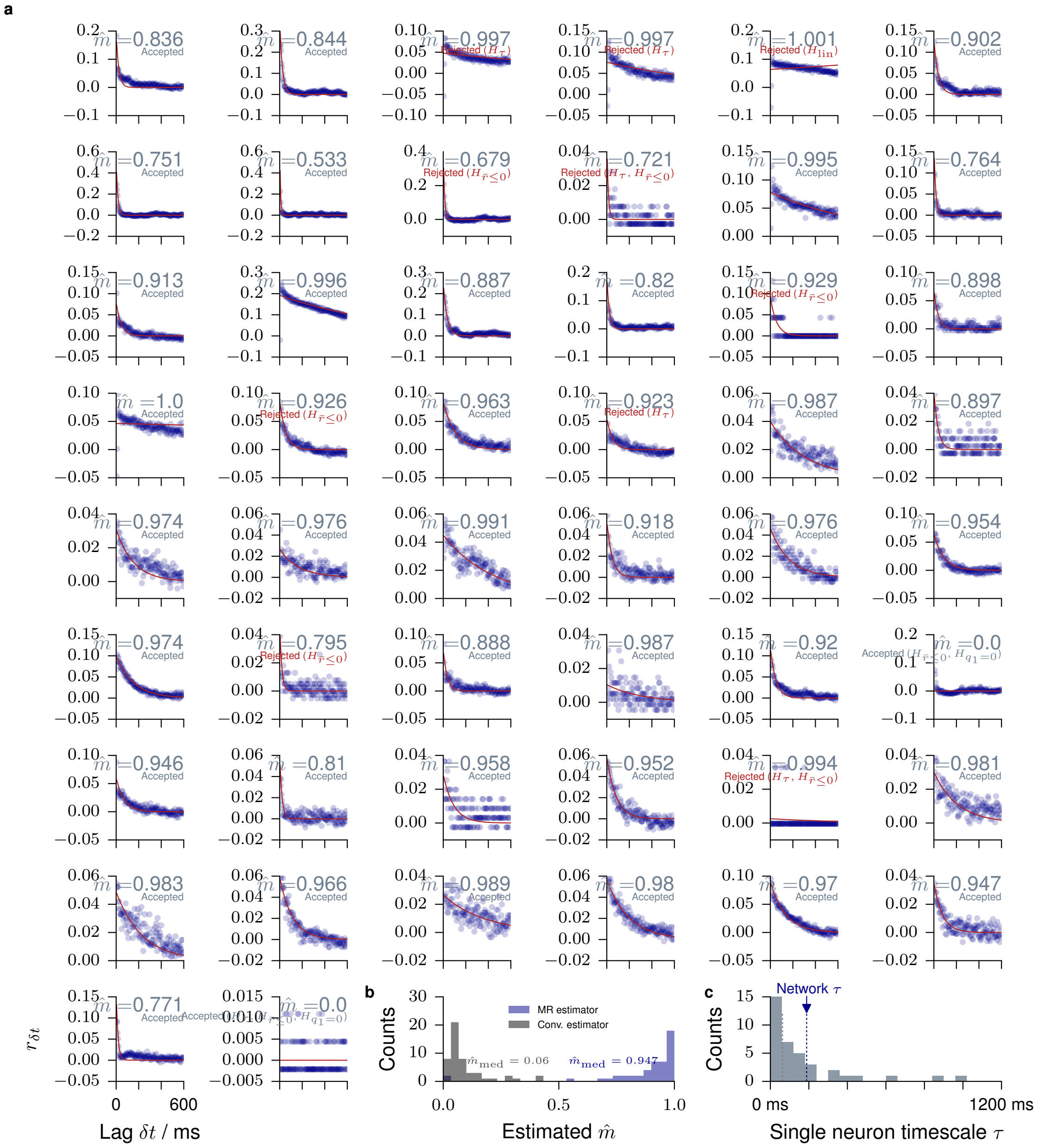

Figure S9: MR estimation from single neuron activity (cat). Modified from Wilting and Priesemann (2018). MR estimation is used to estimate $\hat{m}$ from the activity a_t of a single units in cat visual cortex. **a.** Each panel shows MR estimation for one of the 50 recorded units. Autocorrelations decay rapidly in some units, but long-term correlations are present in the activity of most units. The consistency checks are detailed in Wilting and Priesemann (2018). **b.** Histogram of the single unit branching ratios $\hat{m}$, inferred with the conventional estimator and using MR estimation. The difference between these estimates demonstrates the subsampling bias of the conventional estimator, and how it is overcome by MR estimation. **c.** Histogram of single unit timescales with their median (gray dotted line) and the timescale of the dynamics of the whole network (blue dotted line).

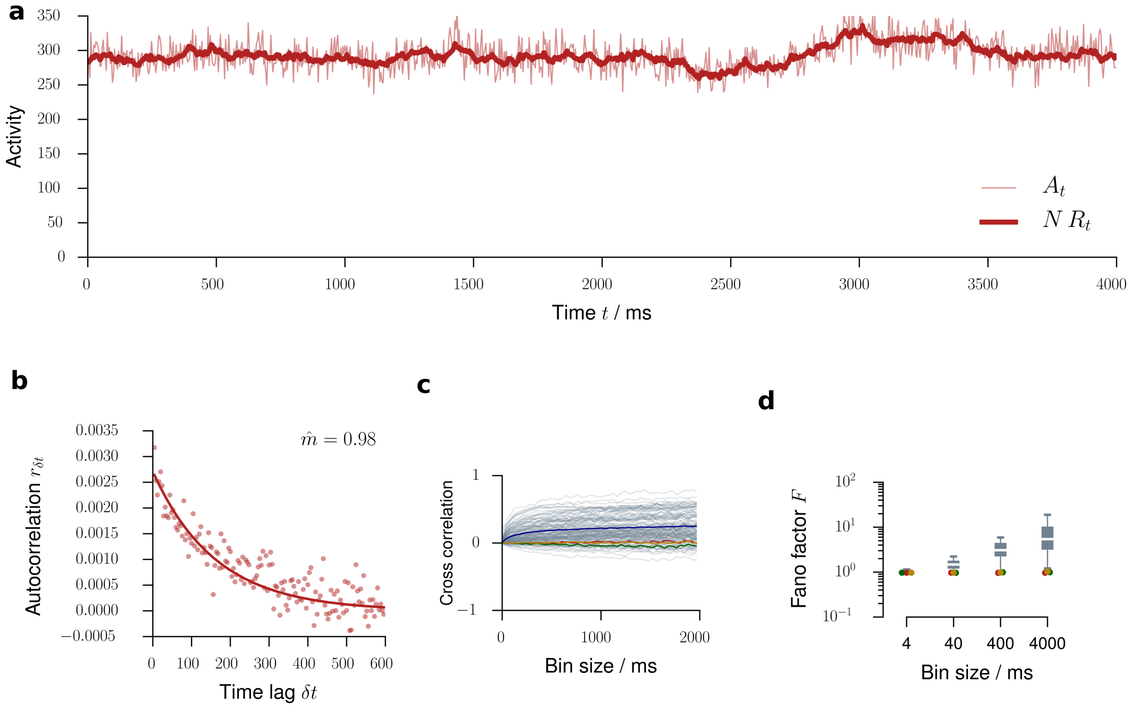

Figure S10: Doubly stochastic model. Instead of a branching model, we here matched a doubly stochastic process to the data. The rates evolved according to $R_{t+1} = m\,R_t + h_t$ where h_t is drawn from a Poisson distribution. The actual activity is then drawn from a Poisson distribution according to $A_t \sim \mathrm{Poi}(N\,R_t)$. Here, results for the experiment in cat visual cortex are shown. **a** Time evolution of R_t and A_t. As the activity is not fed back into the evolution of R_t, the second step effectively adds measurement noise to the underlying process. **b** The subsampled activity (50 out of 10,000, as in the branching models) shows the expected autocorrelation function. **c** Any of the doubly stochastic processes underestimated the spike count cross correlations. **d** Any of the doubly stochastic processes underestimated the single unit Fano factors.

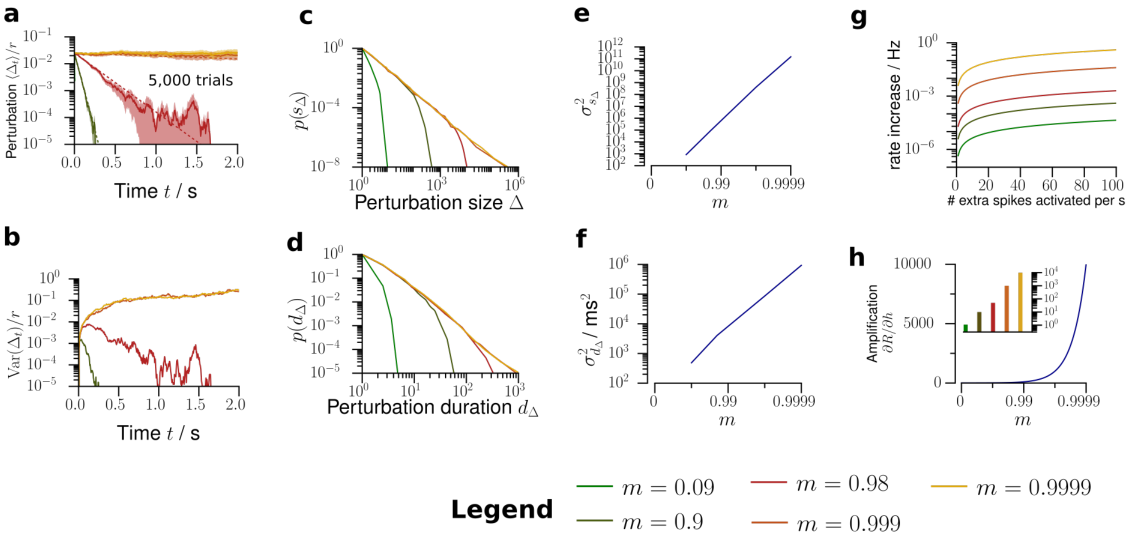

Figure S11: Further predictions about network activity. **a**. The model predicts that the perturbation decays exponentially with decay time $\tau = -\Delta t/\log m$. **b** The variance across trials of the perturbed firing rate has a maximum, whose position depends on m. **c**. Depending on m, the model predicts the distributions for the total number of extra spikes s_Δ generated by the network following a single extra spike. **d**. Likewise, the model predicts distributions of the duration d of these perturbations. **e**. Variance of the total perturbation size as a function of m. **f**. Variance of the total perturbation duration as a function of m. **g**. Increase of the network firing rate as a function of the rate of extra neuron activations for different m. **h**. Amplification (susceptibility) $\mathrm{d}r/\mathrm{d}h$ of the network as a function of the branching ratio m.
